# Differential Effect of Actomyosin Relaxation on the Dynamic Properties of Focal Adhesion Proteins

**DOI:** 10.1371/journal.pone.0073549

**Published:** 2013-09-09

**Authors:** Irena Lavelin, Haguy Wolfenson, Israel Patla, Yoav I. Henis, Ohad Medalia, Tova Volberg, Ariel Livne, Zvi Kam, Benjamin Geiger

**Affiliations:** 1 Department of Molecular Cell Biology, Weizmann Institute of Science, Rehovot, Israel; 2 Department of Neurobiology, Tel Aviv University, Tel Aviv, Israel; 3 Department of Life Sciences and the National Institute for Biotechnology in the Negev (NIBN), Ben-Gurion University of the Negev, Be’er-Sheva, Israel; 4 Department of Biochemistry, University of Zurich, Zürich, Switzerland; The Beatson Institute for Cancer Research, United Kingdom

## Abstract

Treatment of cultured cells with inhibitors of actomyosin contractility induces rapid deterioration of stress fibers, and disassembly of the associated focal adhesions (FAs). In this study, we show that treatment with the Rho kinase inhibitor Y-27632, which blocks actomyosin contractility, induces disarray in the FA-associated actin bundles, followed by the differential dissociation of eight FA components from the adhesion sites. Live-cell microscopy indicated that the drug triggers rapid dissociation of VASP and zyxin from FAs (τ values of 7-8 min), followed by talin, paxillin and ILK (τ ~16 min), and then by FAK, vinculin and kindlin-2 (τ = 25-28 min). Examination of the molecular kinetics of the various FA constituents, using Fluorescence Recovery After Photobleaching (FRAP), in the absence of or following short-term treatment with the drug, revealed major changes in the k_on_ and k_off_ values of the different proteins tested, which are in close agreement with their differential dissociation rates from the adhesion sites. These findings indicate that mechanical, actomyosin-generated forces differentially regulate the molecular kinetics of individual FA-associated molecules, and thereby modulate FA composition and stability.

## Introduction

Integrin-mediated cell-extracellular matrix (ECM) adhesions play key roles in tissue formation and morphogenesis, and in the generation and transmission of adhesion-dependent signals [1–3]. Recent studies indicate that the integrin family of matrix adhesions is highly heterogeneous, displaying conspicuous variations in overall structure, subcellular distribution, and specific molecular composition [1,4,5]. Consequently, different adhesions display diverse functional properties, including selective binding to the ECM, and a differential capacity to sense its mechanical properties and to actively remodel it [6–8].

Live-cell microscopy of cells tagged with specific focal adhesion (FA) components demonstrated that integrin adhesions are dynamic structures that undergo major morphological transformation during their formation and maturation, initially forming nascent adhesions, mainly along the leading lamellae, and later expanding into large focal adhesions, typically several square micrometers in size, that are associated with actomyosin-rich stress fibers [9–12]. Depending on the cell type and ECM properties, these FAs can induce ECM fibrillogenesis and transform into fibrillar adhesions [13,14]. These transformations were shown to be highly mechanosensitive processes; thus, the formation and stability of FAs depend on contractile forces generated by the associated actin cytoskeleton. Inhibition of these contractions (e.g., by Rho-kinase or specific actomyosin inhibitors) leads to FA dissociation, and to disruption of the associated stress fibers [15–19]. At the same time, it was shown that myosin II-independent integrin adhesions also exist, and their properties were characterized [20,21].

The molecular composition and nano-architecture of FAs are believed to play key roles in regulating the diverse scaffolding and signaling activities of cells; yet the molecular mechanisms underlying these processes are still largely unclear. Attempts to characterize the molecular composition of integrin adhesions revealed a rich variety of “adhesome” molecules (over 200 components known at present) that collectively perform and regulate the various scaffolding and signaling functions of these adhesions [10,22]. Among them are membrane receptors, adaptor molecules, and cytoskeleton-associated proteins, which collectively bridge between the ECM and the F-actin cytoskeleton. Additional regulatory molecules, including diverse kinases, phosphatases, and G-protein regulators, participate in both modulation of the adhesions, and in integrin-mediated signaling processes that affect cell behavior and fate [10,22].

In this study, we tested the hypothesis that variations in the mechanical force applied to FAs by means of the cellular contractile machinery, differentially affect the binding and dissociation of various adhesome components and, hence, modulate FA composition, molecular architecture and, eventually, function. Specifically, we examined how inhibition of actomyosin contractility affects the association of different FA components with the adhesion sites, by quantifying temporal changes in the levels and organization of eight different adhesome proteins, following treatment with the Rho-kinase inhibitor Y-27632. We demonstrate here that the components tested dissociate from FAs at differing rates, accompanied by major structural changes in the FA-associated cytoskeleton, as revealed by cryo-electron tomography. We further show that the differential dissociation of the tested proteins can be attributed to specific changes in their k_on_ and k_off_, values, induced by the drug. Calculation of the expected dissociation rate of each molecule from FAs in treated cells, based on these kinetic changes, accurately fit the dissociation values measured by Fluorescence Recovery After Photobleaching (FRAP) microscopy. Furthermore, we show that Y-27632-treated cells can still form and maintain modified integrin adhesions with the underlying substrate. Notably, such adhesions, formed around the cell center, predominantly contain ILK, kindlin-2 and paxillin, but little or no zyxin, VASP, talin, vinculin and FAK, while those formed at the cell periphery are similar to those of untreated ones, except that they are largely devoid of VASP and zyxin.

## Materials and Methods

### Cell culture, DNA constructs, and reagents

HeLa JW cells and rat embryo fibroblast cell line (REF-52) cells (generously provided by the Cold Spring Harbor Laboratory [23]) were cultured in Dulbecco’s modified Eagle’s medium (DMEM) containing 10% fetal bovine serum (FBS), 2 mM glutamine and 100 U/mL penicillin-streptomycin in a 5% CO_2_ humidified atmosphere, at 37°C. All cell culture reagents were purchased from Biological Industries, Ltd. (Beit Haemek, Israel), and used according to the manufacturer’s instructions. Transfections employing plasmid DNA were carried out using a jetPEI DNA transfection reagent (Polyplus Transfection, Illkirch, France). Expression constructs used in this study included: EGFP-tagged vinculin and paxillin [24]; GFP-zyxin, a gift from Jurgen Wehland, the Gesellschaft für Biotechnologische Forschung, Braunschweig, Germany (Jurgen passed away on August 16, 2010); EGFP-VASP, talin and FAK (obtained from Michael W. Davidson, Florida State University, Tallahassee, FL, USA); and EGFP-ILK and EGFP- 2 (kindly provided by Reinhard Fässler, Max Planck Institute, Martinsried, Germany). For all live-cell imaging experiments, cells were plated on glass coverslips coated with fibronectin (FN) (20 µg/ml, Sigma-Aldrich, Rehovot, Israel). ROCK inhibitor Y-27632 (Calbiochem, San Diego, CA, USA) was used at a concentration of 10 µM.

### Immunofluorescence staining, immunofluorescence microscopy and image analysis

For immunostaining, cells cultured on glass coverslips were fixed/permeabilized for 2 min in phosphate-buffered saline (PBS) containing 0.5% Triton X-100 and 3% paraformaldehyde, and post-fixed with 3% paraformaldehyde in PBS for 30 min. The cells were stained with primary antibodies for 1 h at room temperature, washed in PBS, incubated for 1 h with fluorophore-conjugated secondary antibodies (Jackson ImmunoResearch Laboratories, Inc., West Grove, PA, USA), washed again, and mounted in Elvanol (Moviol 4–88; Hoechst, Frankfurt, Germany). Images were acquired using the DeltaVision RT microscopy system (Applied Precision Inc., Issaquah, WA, USA). Primary antibodies used in this study included: mouse anti–paxillin and mouse anti-FAK (BD Transduction Laboratories, San Jose, CA, USA); mouse anti-ILK (Santa Cruz Biotechnology, Santa Cruz, CA, USA); mouse anti-vinculin (Sigma-Aldrich); and mouse anti-VASP, anti-talin and rabbit anti–kindlin 2 (Abcam, Cambridge, UK). Phalloidin labeled by FITC, TRITC or coumarin, for F-actin staining, was obtained from Invitrogen (Carlsbad, CA, USA). Other antibodies were prepared by the Antibody Production Laboratory of the Department of Biological Services, Weizmann Institute of Science: (http://bioservices.weizmann.ac.il/antibody/about.html).

TIRF images were acquired using the DeltaVision Elite microscopy system equipped with a multi-line TIRF module (Applied Precision, Inc.), and time-lapse movies were taken at 3-min intervals, unless otherwise indicated, over a period of 2 h, using the same system. Quantification of fluorescence intensity and FA area was performed using image analysis software developed in-house, within the UCSF Priism environment (http://msg.ucsf.edu/IVE/), after correcting for fluorescence bleaching. To segment FAs, local background was subtracted and a watershed algorithm was applied, to separate neighboring adhesions. Quantification enabled the definition of ~30 geometric and intensity features for each adhesion site, including dimension, shape and intensity. The intensity and lifespan of individual FAs as a function of time were analyzed, following tracking of individual FAs. For fluorescence ratio imaging, cells were double-labeled for zyxin and vinculin, and the intensity ratio was computed per pixel, as previously described [24].

### Fluorescence Recovery After Photobleaching (FRAP) assay and FRAP data analysis

FRAP studies were conducted on HeLa cells expressing GFP-tagged FA proteins, 24 h after plating on FN-coated glass coverslips [25,26]. Measurements were taken at 37°C in Hank’s Buffered Salt Solution (HBSS) supplemented with 20 mM HEPES, pH 7.2. An argon ion laser beam (Innova 70C; Coherent, Palo Alto, CA, USA) was focused through a fluorescence microscope (AxioImager D.1; Carl Zeiss MicroImaging, Oberkochen, Germany) to a Gaussian spot of 0.77±0.01 µm (with a plan-apochromat 63x/1.4 NA oil-immersion objective). After a brief measurement at monitoring intensity (528 nm, 1 µW), a 5 mW pulse (~10 ms) was used to bleach 50–75% of the fluorescence in the spot, after which the monitoring intensity beam was used to track the recovery of fluorescence over time.

To reduce the noise present in single FRAP curves, each curve was normalized by setting the pre-bleach intensity to 1, and 20-30 curves were then averaged, starting from the bleach point for synchronization. The resulting curve was fitted to the mathematical expression describing the full diffusion-exchange model, to extract the k_on_ and k_off_ values for each protein [27]. To increase the stability of the fitting procedure, some of the parameters were introduced as pre-determined, fixed inputs: D_C_ (the diffusion coefficient in the juxtamembrane cytoplasm surrounding the FAs [26]), N_FA_ (number of molecules in the FA), and N_C_ (number of molecules in the juxtamembrane cytoplasm surrounding the FA). To determine D_C_ for each protein, we performed FRAP measurements on FAs at a short timescale (2-3 s; data not shown), where the contribution of slow exchange is negligible, and fit the data as in [25–27]. For all proteins, these measurements yielded D_C_ values of 1-1.5 µm^2^/s. For N_FA_ and N_C_, we first estimated their values by measuring the fluorescence in a similarly sized region at an FA, as well as immediately adjacent to it, and then verified these estimates by fitting the FRAP data to the full model, with N_FA_ and N_C_ set as fitting parameters. Finally, we fitted the data to the full model, in which the D_C_, N_FA_, and N_C_ values that were extracted as described above, were entered as fixed input. In all cases, the values of D_M_ (the diffusion coefficient inside the FA) were negligible, relative to the fluorescence recovery time (i.e., <0.01 µm^2^/s).

Since both k_on_ and k_off_ were extracted in this manner, we could use these values to calculate, for each protein, the theoretical dissociation rate from the adhesion. For an adhesion to disassemble, the outward flux of proteins must be greater than the inward flux. The turnover of proteins in adhesions, at steady state, is a first-order process, i.e., for a protein to enter the adhesion, a bound protein must first detach from its binding site. We propose that during adhesion disassembly, the available binding sites for proteins from the cytoplasm are gradually reduced as a result of loss in tension; namely, after a specific protein molecule exits its binding site [25], the conformation of this site changes, thereby blocking rebinding of the cytoplasmic molecule. Since the “on rate” depends on the number of available binding sites, which changes over time at an unknown rate, the measured “on rate” constant is an apparent k_on_. As a first approximation, and for the purpose of comparing the different plaque proteins on both molecular and structural scales during adhesion disassembly, we defined: K_dis_ = k_off_ -k_on_. We then generated a theoretical disassembly curve for each protein, using: y=e^−K_dis_*t^, assuming that FA disassembly behaves as a mono-exponential process [28].

### Cryo-electron tomography, correlative micros y=e^−K_dis_*t^ copy, and image processing

Carbon-coated 200-mesh gold grids (Quantifoil, Micro Tools GmbH, Jena, Germany) were rinsed in PBS and overlaid on a drop of 50 µg/ml FN for 45 minutes. REF52 cells expressing YFP-paxillin were applied to the grids in concentrations of 100 cells per grid, and cultured for 24 h. As previously shown [29], these cells form FAs that are compatible with cryo-electron tomography (cryo-ET). A 5 µl drop of BSA-coated 15 nm gold colloids in PBS was added to the grids before plunging them into liquid nitrogen-cooled ethane, as described in [30]. Specimens were then transferred into a 300 FEG Polara microscope (FEI, Eindhoven, The Netherlands) equipped with a Gatan post-column GIF 2002 energy filter, and tilt series were collected, typically covering an angular range of -60° to 66°, sampled in 2° tilt increments, and at a 14 µm underfocus. Pixel size was 0.82 nm at the specimen level.

Projection images (2048 x 2048) were aligned to a common origin, using gold fiducial markers, and reconstructed by means of weighted back-projection, as implemented by means of a TOM toolbox software package [31]. All tomograms were reconstructed with a binning factor, to yield a 1.64 nm voxel size. For visualization purposes, the reconstructed volumes were processed by an anisotropic de-noising algorithm [32]. Individual adhesion complexes were identified and extracted, *in silico*. Two-dimensional images of these elements were calculated by projecting the volumes (32 x 32 x 11 voxels) along the Z axis, using the EM software package [33]. The particle stack was then masked and normalized prior to PCA, and followed by K-means classification (SPIDER). Class averages containing many features were chosen to be the first references for a multi-reference alignment of the data set. This strategy was iterated for five rounds until no major changes occurred in the classes, and in the alignment of single images.

### Immunoblotting

HeLa JW cells were lysed with radioimmune precipitation assay buffer (1% NP-40, 1% sodium deoxycholate, 0.1% SDS, 150 mM NaCl, 50 mM Tris, pH 8.0) containing a protease inhibitor cocktail from Roche Applied Science (Indianapolis, IN, USA). Protein extracts were subjected to 8% SDS-PAGE, transferred to a nitrocellulose membrane (Whatman, Dassel, Germany), and probed with polyclonal anti-phospho-myosin light chain (Ser19) (Cell Signaling Technology, Beverly, MA, USA), anti-myosin light chain (Cell Signaling Technology) and anti-β-tubulin (Sigma-Aldrich, Rehovot, Israel) primary antibodies.

## Results

### Differential dissociation of adhesome components from FAs upon Y-27632 treatment

**Figure 1:**
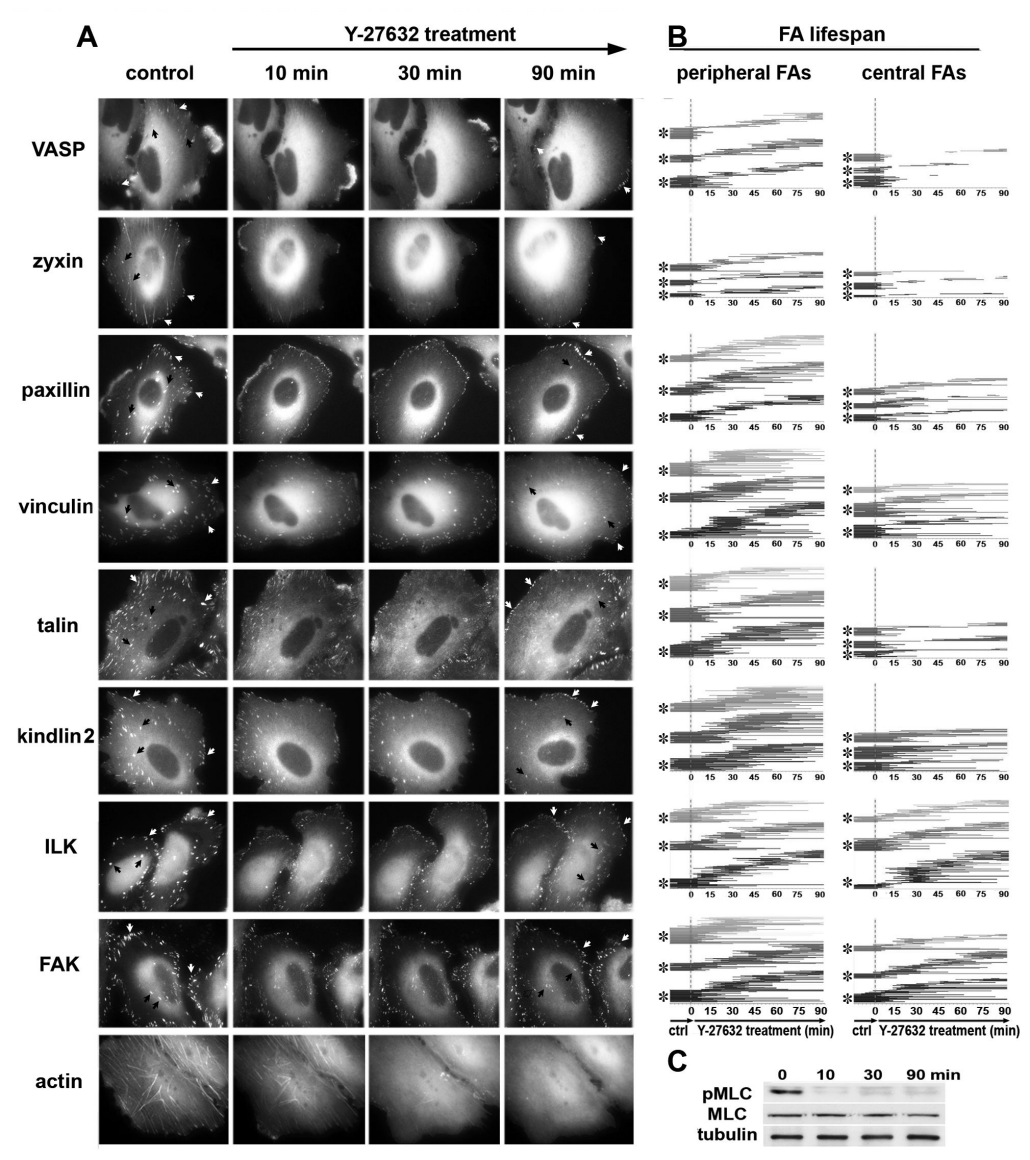
Effect of Y-27632 treatment on FA reorganization in HeLa JW cells. (**A**) Localization of GFP-tagged VASP, zyxin, paxillin, vinculin, talin, kindlin-2, ILK and FAK in untreated cells (control) or in the cells treated with 10 µM Y-27632 for 10, 30 or 90 min (shown are snapshots from time-lapse movies). White and black arrowheads point to peripheral and central adhesions, respectively. (**B**) FA lifespan in three different cells tagged by each of the tested proteins prior to and during Y-27632 treatment, as tracked by time-lapse video microscopy. Each line marks the lifespan of an individual adhesion site, from its formation until its complete disassembly. Individual cells are marked by asterisks on the Y axes. The vertical dashed line marks the beginning of the Y-27632 treatment. Peripheral and central FAs were analyzed separately. Note the decay of existing FAs and formation of novel FAs during Y-27632 treatment. (**C**) Western blots showing MLC and pMLC in untreated cells, or in cells treated with 10 µM Y-27632 for 10, 30 and 90 min Tubulin was used here as a loading control.

To explore the differential molecular sensitivity of adhesome molecules to relaxation of actomyosin-generated forces, we expressed, in HeLa JW cells, eight distinct, GFP-tagged FA-associated proteins, and examined the temporal effects of treatment with the Rho-kinase inhibitor Y-27632 on the levels of each, in association with FAs. Specifically, in this study we used quantitative live-cell imaging to examine the drug’s effect on zyxin, VASP, vinculin, paxillin, talin, ILK, kindlin-2, and FAK. In Figure 1A, representative images from time-lapse movies, corresponding to pre-treatment (control), and to 10, 30 and 90 minutes of treatment, are shown.

**Figure 2:**
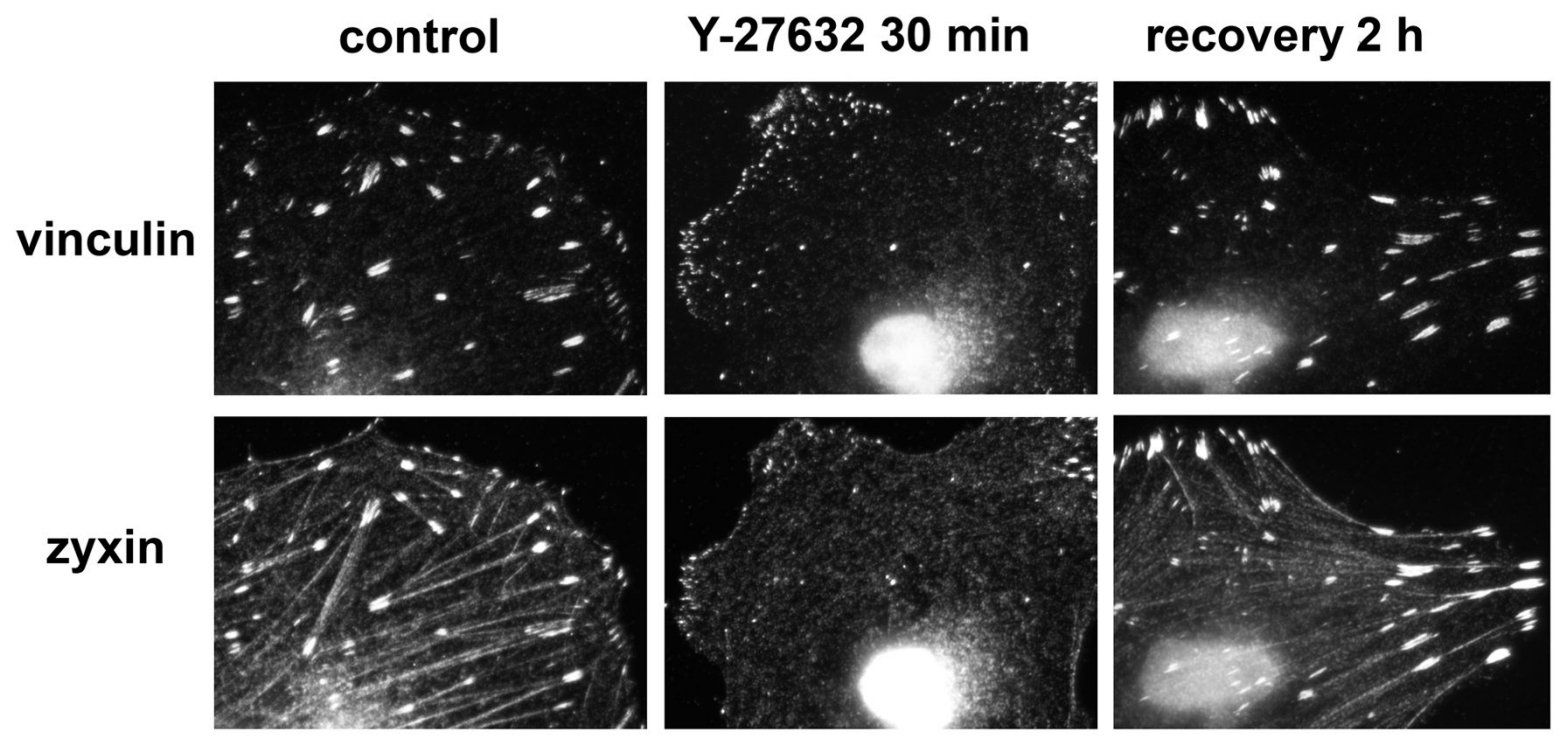
Reversibility of the effect of Y-27632 on cell morphology. Images of Y-27632-treated HeLa JW cells were taken prior to addition of the drug, after 30 min incubation with 10 µM Y-27632, and 2 h after the drug was washed off. The effect of Y-27632 on cell morphology was reversible, as cells regained normal morphology within 2 hours of reagent withdrawal.

To exclude the possibility that the apparent changes in protein distribution are affected by the expression of the exogenous, tagged protein, we validated all live-cell data by immunofluorescence labeling of the corresponding endogenous proteins in non-transfected cells at several time points, following the same treatment (data not shown). Upon longer exposure to Y-27632, *de novo* formation of matrix adhesion sites occurred throughout the entire ventral cell membrane (Figure 1B), despite the complete inhibition of MLC phosphorylation (Figure 1C). Notably, all these effects were reversible, as cells regained normal morphology within 2 hours of Y-27632 withdrawal (Figure 2).

**Figure 3:**
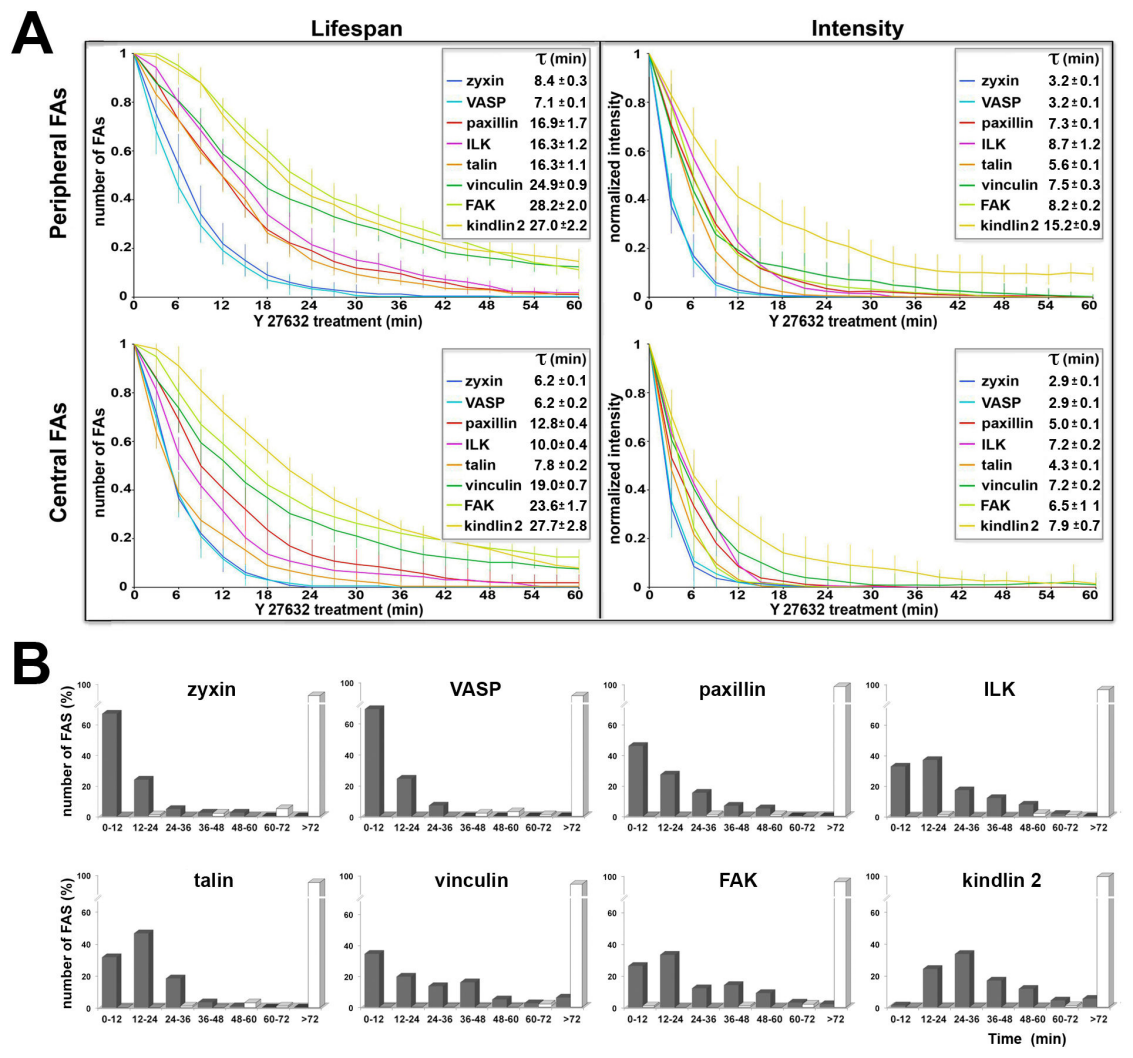
Differential dissociation rates of adhesome components in cells treated with Y-27632. (**A**) FA lifespan (expressed as the relative number of surviving FAs) and average normalized fluorescence intensity for each protein, in peripheral and central FAs separately, as tracked by time-lapse video microsopy for a hundred individual FAs. τ values were calculated by fitting the individual decay profiles to a single-exponential function. Note that the order of exit of the different molecules is the same in all graphs, with zyxin and VASP as the first to leave and kindlin 2 as the last. (**B**) Based on the same dataset shown in **A**, the differential effect of Y-27632 on the lifespan of the FA proteins tested, in peripheral FAs, is shown for each protein, separately. FA lifespan for each protein is expressed as the percentage of total FA number over time. Untreated cells are shown as white columns; cells treated with Y27632 as black columns.

Closer inspection of the treated cells indicated that FAs located within the lamellipodium or the lamella responded quite differently than those located around the cell center. In view of these differences (for details, see below), we quantified the responses of FAs located in these two subcellular regions (namely, “peripheral FAs” and “central FAs”) separately. Examination of the differential effect of Y-27632 on the FA components tested, in both peripheral and central adhesions, indicated that the different proteins dissociate from FAs at distinct rates (Figure 3A), as previously noted for some of these molecules [25,34,35]; see also Discussion.

To further quantify FA turnover, we then calculated FA lifespan (from “birth” to “death”) for each of the studied proteins, as well as the exponential time-constant (τ) and fluorescence intensity of individual adhesion sites, during Y-27632 treatment (Figure 3A for FA number and intensity, and 3B for FA lifespan, sorted by protein). The decay profiles are well described by a single-exponential curve. The time-constant (τ) extracted from curve fitting the data (Figure 3A), provides a natural physical time scale, as the system relaxes to 1/e (~37%) of its initial value. It is noteworthy that in untreated HeLa JW cells, the apparent τ of FAs labeled by all of the adhesome components tested varies, usually ranging between a few tens of minutes to a few hours (data not shown).

Quantification of the decline in the number and intensity of the initial peripheral and central adhesions, following Y-27632 treatment (Figure 3A), indicates that Y-27632 dramatically reduced the lifespan of all existing FAs (both “peripheral” and “central”), though different proteins dissociate from FAs at varying rates. The first molecules to leave FAs were zyxin and VASP, with τ values of 7-8 min; talin, paxillin and ILK dissociate from FAs at a slower rate (τ ~16 min); while FAK, vinculin and kindlin-2 showed the slowest dissociation dynamics, with τ values of 25-28 min (see inserts in Figure 3A, values for the lifespans of peripheral FAs). To further emphasize the vast differences between the lifespans of FAs containing the different FA proteins, we constructed a histogram (Figure 3B), depicting the lifespans of Y-27632-treated peripheral adhesions. Comparison of peripheral and central adhesions indicated that the latter dissociated at a 1.3-twofold faster rate; yet the order of exit of the different molecules was essentially the same.

### Effect of Y-27632 treatment on FA nano-architecture

**Figure 4:**
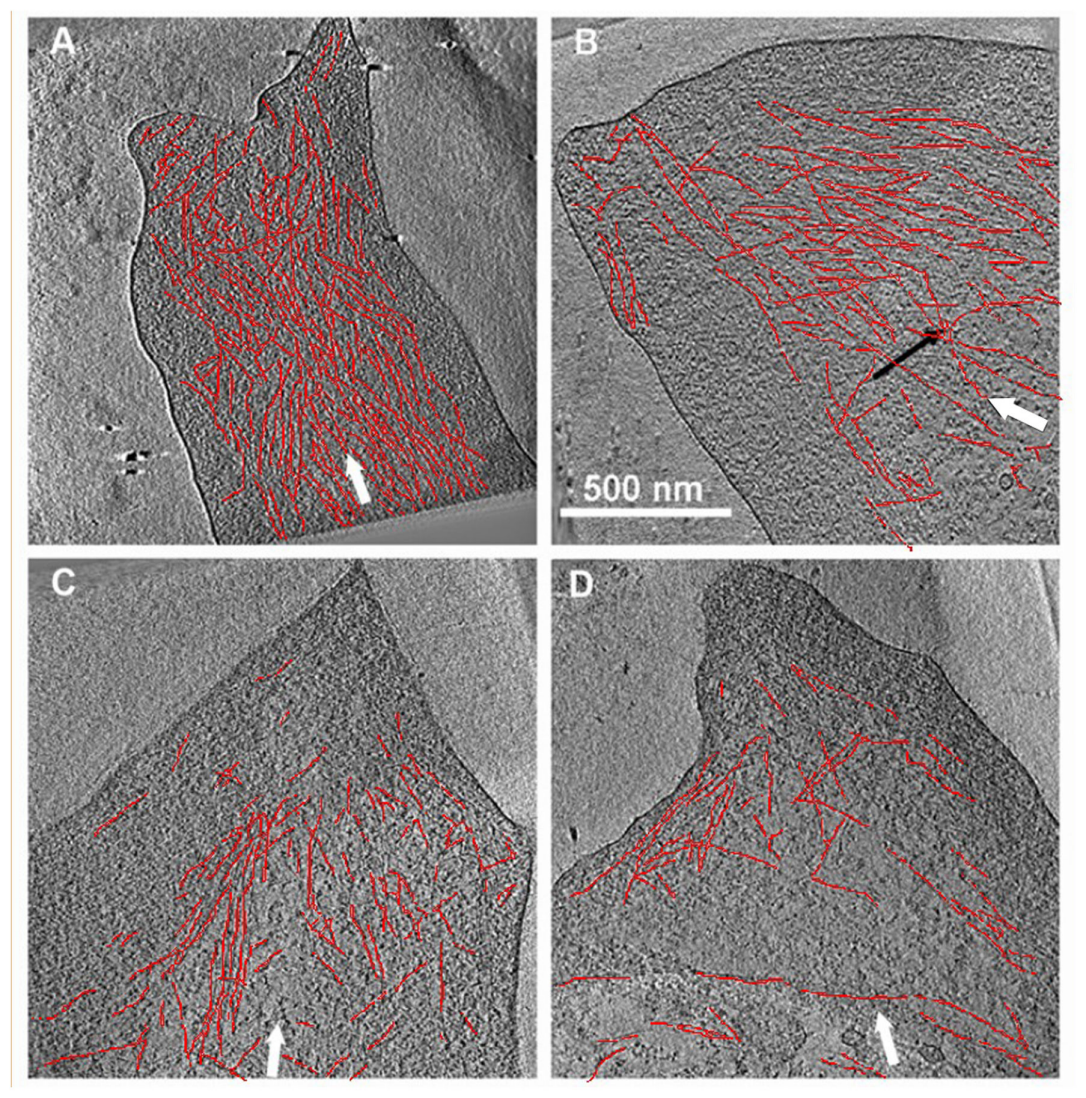
Cryo-electron tomography of cells incubated with Y-27632. REF52 cells expressing YFP-paxillin growing on EM grids, untreated (**A**) or treated for 3 (**B**), 10 (**C**) or 30 (**D**) minutes with Y-27632, were analyzed by correlative (fluorescence-cryo-electron tomography) microscopy. Individual FAs, identified by light microscopy, were subjected to cryo-ET and 3D image reconstruction. The major axis of each FA is indicated by a white arrow; some of the more prominent filaments are highlighted by a thin red **line**. Ten nm-thick slices through the cryo-tomograms of representative FAs indicated a major reduction in actin alignment, already observed following 3 min treatment with the drug (compare B to A: The black arrow, in B, points to an example of misaligned filaments, commonly seen after Y-27632 treatment). Upon longer treatment with Y-27632, progressive misalignment of actin filaments is apparent, accompanied by a marked reduction in filament density. The scale bar in B represents 500 nm.

Examination of FAs of Y-27632-treated REF52 cells by means of cryo-ET revealed substantial changes in the molecular architecture of the cell-matrix adhesions (Figure 4, and Figure S1). REF52 cells were chosen for these experiments since they are thin, form FAs that are suitable for cryo-ET [29], and the spatio-temporal dynamics of their FAs in the presence or absence of Y-27632 are similar to those of HeLa cells (Figure S2). Image reconstructions of FAs after 3, 10 and 30 minutes of incubation with Y-27632 (Figure 4B, C and D, respectively) show major structural alterations in the adhesion machinery, compared to untreated cells (Figure 4A). These alterations manifest themselves in disorganization of the FA-associated actin network (see also 29) with a major loss of aligned filaments, already detected after 3 min of incubation with the drug (Figure 4B). Upon longer treatment (10-30 minutes), the filaments become increasingly disorganized and sparse, as shown in Figure 4D. These early alterations of FA-associated actin filamentsoccur before a major decline in the levels of plaque proteins is evident, suggesting that loss of actin alignment in FAs constitutes an early response to Y-27632 treatment.

### The nature of the adhesion structures formed in the presence of Y-27632

**Figure 5:**
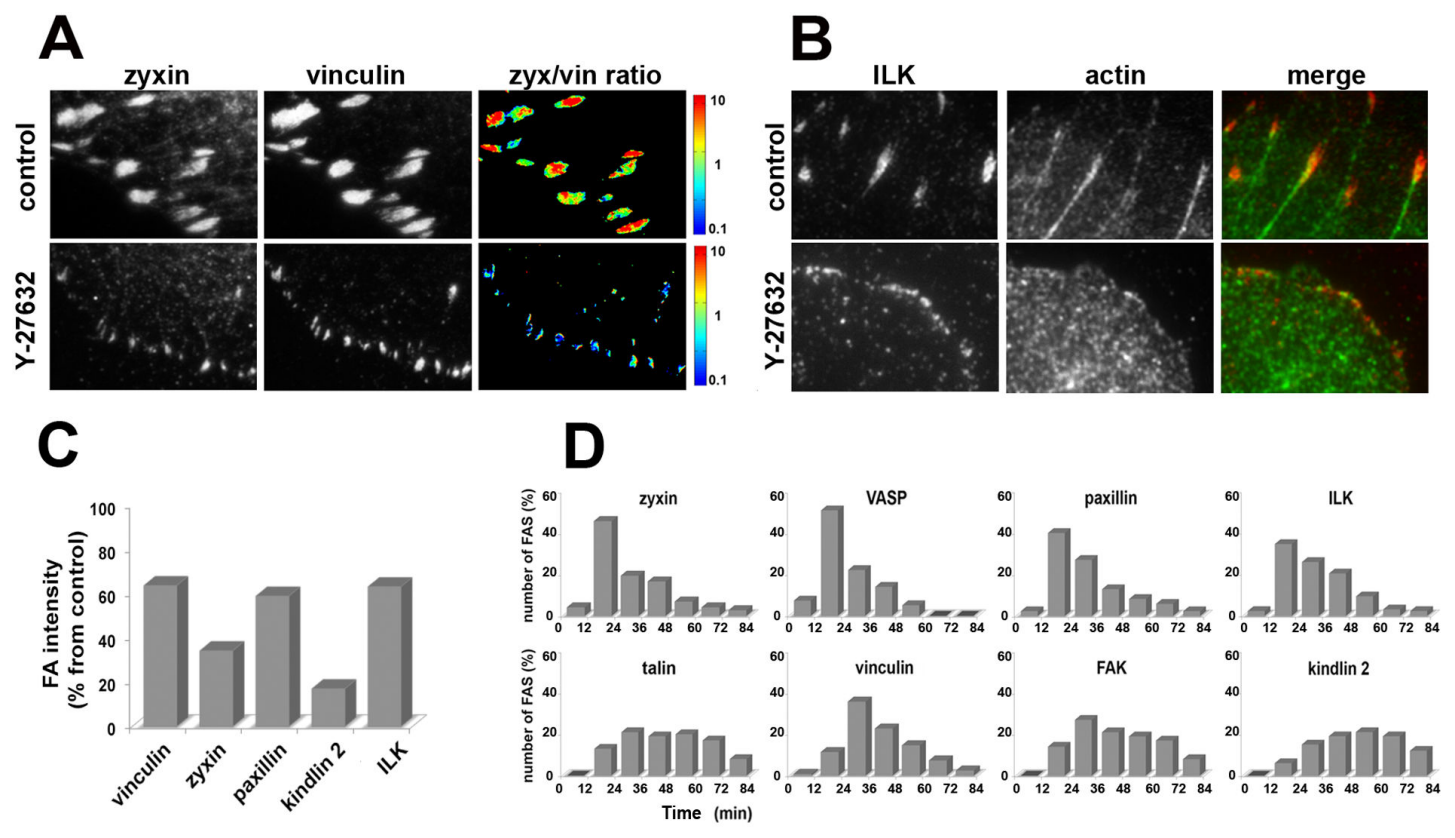
Peripheral FAs in Y-27632 treated cells differ from control FAs in molecular composition and lifespan. (**A**) Comparison of zyxin and vinculin fluorescence intensity for FAs in untreated cells, and peripheral FAs in Y-27632 treated cells. Cells prior to and following 60 min of Y-27632 treatment were double-labeled for zyxin (left) and vinculin (center). **Right**: The fluorescence ratio image (zyxin/vinculin) in a logarithmic, blue-to-red spectrum scale, representing the ratio value. (**B**) Comparison of ILK and actin localization in FAs of untreated cells, and in peripheral FAs of Y-27632 treated cells. Two-color TIRF microscopy of the cells prior to and following 60 min of Y-27632 treatment was used to assess for ILK (left) and actin staining (center). **Right**: A merged image (ILK in red and actin in green), demonstrating enrichment of actin in the FA sites. (**C**) Normalized vinculin, zyxin, paxillin, kindlin-2 and ILK fluorescence intensities in peripheral FAs following 60 min of Y-27632 treatment, expressed as a percentage of intensity of the same protein in FAs of untreated cells. Fluorescence intensity measurements were averaged over 150 individual FAs. (**D**) Lifespan of peripheral FAs for each protein, expressed as the percentage of total FA number over time, averaged over 150 individual FAs for each protein.

Long-term treatment of cells with Y-27632 enables the *de novo* formation and maintenance of integrin adhesions at the cell periphery and at the cell center, both of which differ from those formed by untreated cells. The new peripheral adhesions are insensitive to Y-27632, smaller than those of untreated cells, but display a protein molecular composition similar to those of untreated cells, yet with reduced levels of zyxin and kindlin-2 (Figure 5A and C). Two-color TIRF microscopy revealed that ILK density, used as a marker for these adhesion structures, correlated with F-actin density at the same locations, suggesting that these structures are associated with actin filaments (Figure 5B). The lifespan calculated for each protein (Figure 5D) is much shorter than that found in untreated FAs, and varies from a few minutes for zyxin and VASP, to half an hour for kindlin-2 (see Figure 3), compared to over 2 hours in untreated cells.

**Figure 6:**
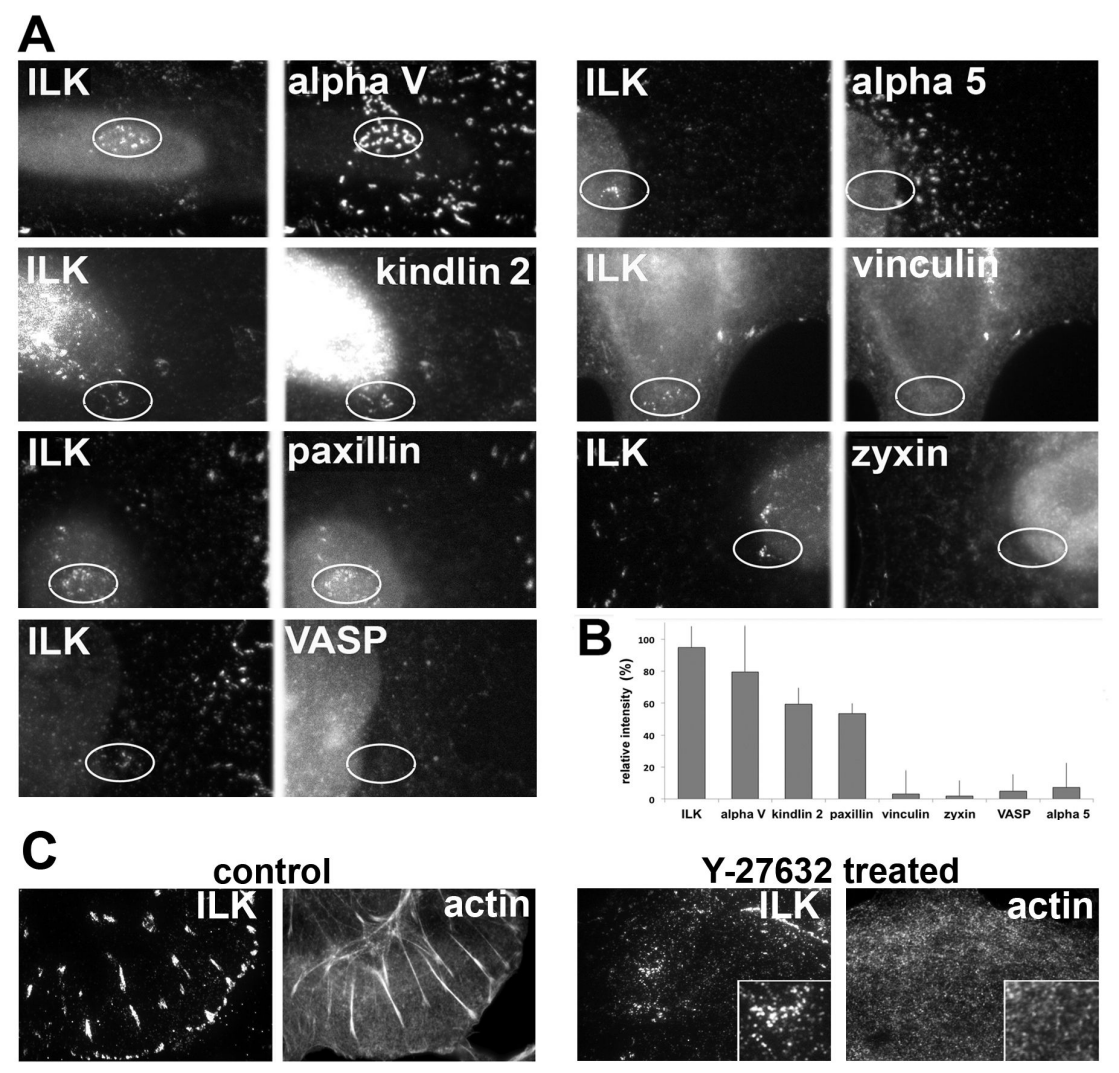
Molecular composition of the novel central adhesions formed following Rho-kinase inhibition. (**A**) HeLa JW cells were treated with 10 µM Y-27632 for 60 min, and double-labeled for ILK, the most prominent component of these adhesions, as well as for additional FA components. The same field is shown for two labeled proteins; circles indicate the region of interest. (**B**) Relative intensity of the novel central adhesions marked by the studied proteins, following 60 min of Y-27632 treatment. The relative intensity was calculated as the percentage of fluorescence intensity measured for the corresponding protein in the untreated FAs. (**C**) Comparison of ILK and actin localization in FAs of untreated cells, and in the central adhesions of Y-27632 treated cells. Two-color TIRF microscopy of the cells prior to (left panel) and following 60 min of Y-27632 treatment (right panel) was used to assess the localization of ILK and actin. No correlation was found between the densities of these two proteins in the central adhesions (see inserts).

The new central adhesions formed in the presence of the drug display a dot-like morphology, and consist of only a subset of the components present in classical FAs: ILK and α_v_ integrin are prominent components, as well as kindlin-2 and low levels of paxillin. Notably, zyxin, VASP, vinculin, talin, FAK and the α_v_β_1_ integrin appear to be absent from these adhesion structures (Figure 6A and B). Furthermore, the ILK-rich patches were clearly visualized by two-color TIRF microscopy, confirming their association with the ventral cell membrane, while F-actin was hardly detectable in the same structures (Figure 6C).

### The molecular mechanism underlying ROCK-dependent modulation of FAs

**Figure 7:**
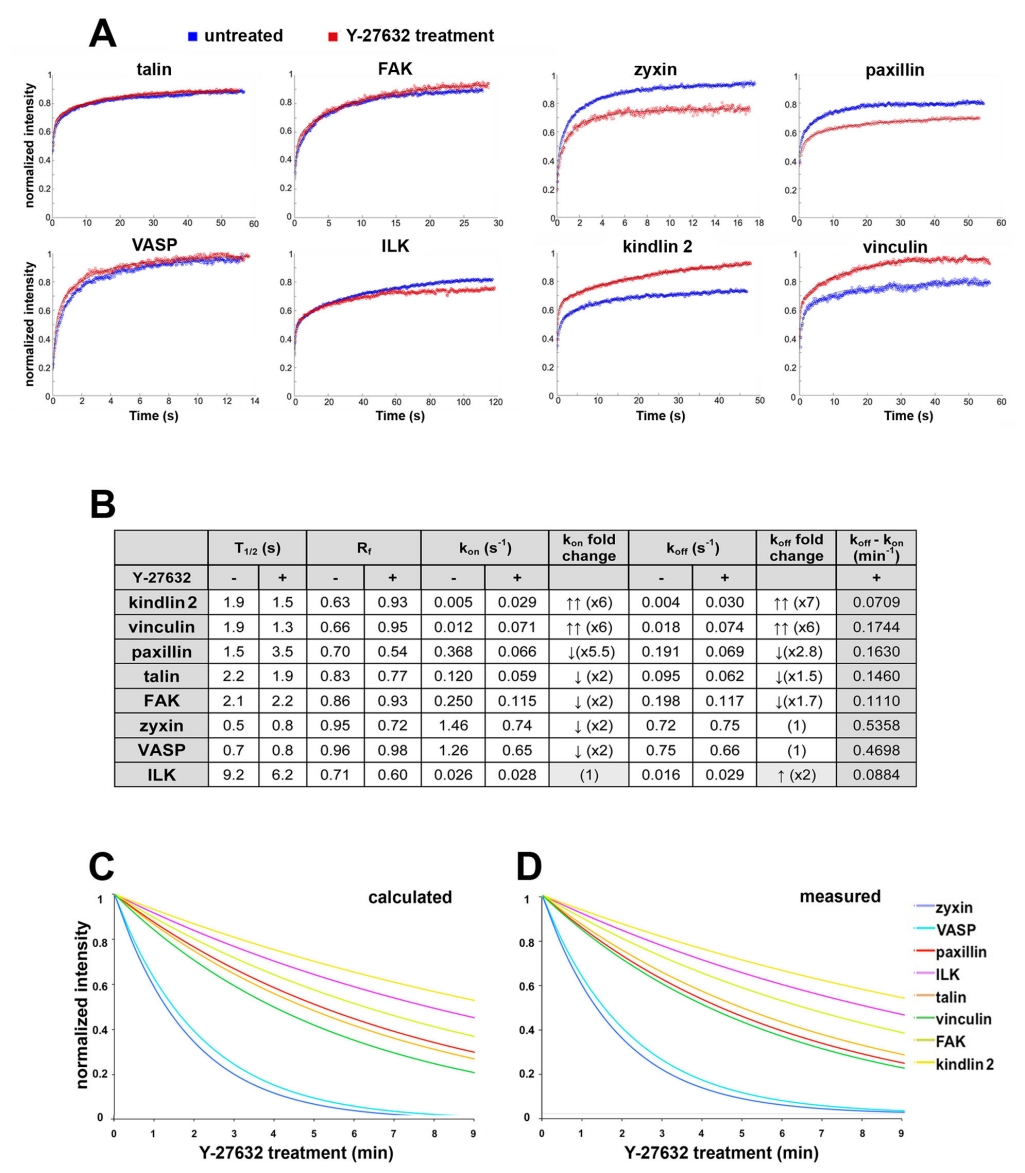
Dynamics of FA plaque proteins following Y-27632 treatment. (**A**) Normalized averaged FRAP curves (n=20-30) of FA plaque proteins in untreated and Y-27632 treated cells. For some proteins, no major changes were observed in the FRAP curves in untreated vs. treated cells (talin, FAK, VASP, ILK), whereas other proteins displayed higher (vinculin, kindlin-2) or lower (paxillin, zyxin) FRAP rates following Y-27632 treatment. (**B**) Kinetic parameters of FA proteins as derived from FRAP measurements within the first 9 min of Y-27632 treatment. The half-time to full recovery (T_1/2_) and mobile fraction (R_f_) values of the FRAP curves prior to and following Y-27632 treatment were not indicative of the overall FA disassembly state. However, the differences between the k_on_ and k_off_ values derived from fitting the FRAP data to the full diffusion-exchange model represent the disassembly rate of FAs for each protein, following Y-27632 treatment. (**C**) Theoretical FA disassembly curves calculated from the differences in the expected disassembly rates of tested proteins from the FRAP data. (**D**) Actual disassembly curves measured directly from time-lapse movies, following treatment of HeLa JW cells with Y-27632. Note the remarkable similarity between the calculated and measured values.

To explore the molecular basis for the differential turnover of adhesome components, we performed FRAP assays for the different FA-associated proteins studied herein, both prior to or within a few minutes of Y-27632 treatment. The results, shown in Figure 7A and 7B, demonstrate that the various proteins tested responded differently to the drug. The FRAP rates and the mobile fractions (R_f_) of VASP, FAK and talin were not affected by Y-27632. Paxillin and zyxin, on the other hand, displayed reduced recovery rates and R_f_ values, while vinculin and kindlin-2 exhibited faster turnover in the presence of the drug. Interestingly, a very limited effect of Y-27632 on recovery rate was initially seen in ILK; yet the mobile fraction was apparently reduced.

Calculation of the kinetic parameters (k_on_ and k_off_), performed for each protein in the presence and absence of Y-27632 (see Materials and Methods), showed distinct changes in the ratio between k_on_ and k_off_ values, for all proteins tested. Kindlin-2 and vinculin showed major increases in both k_on_ and k_off_ values; paxillin, talin and FAK showed minor decreases in both values; zyxin and VASP showed a small decrease in k_on_ only, and ILK showed a slight increase in k_off_ only (see Figure 7B). Notably, in untreated cells, the k_on_ values for all proteins tested were higher than the k_off_ values, whereas following Y-27632 treatment, k_off_ exceeded k_on_ for all these proteins. Addition of blebbistatin, instead of Y-27632, induced similar changes in the kinetic parameters of FA components (data not shown), suggesting that it is the relaxation of actomyosin contractility that triggers the unbinding of proteins from FAs, exceeding their binding rate, and consequently leading to a gradual disassembly of the FA structure.

Based on the differences between the k_on_ and k_off_ values induced by Y-27632, we calculated the expected dissociation rate of each of the tested proteins from the adhesion site (assuming that FA disassembly behaves as a mono-exponential process [28]), and compared the predicted dissociation curves with those directly measured by live-cell microscopy (Figure 7C and D). This comparison revealed a remarkable similarity between the calculated and measured values, suggesting that the basis for the differential dissociation of specific adhesion molecules upon suppression of actomyosin contractility is a protein-specific switch in the relative k_on_ and k_off_ values, induced by the drug.

FRAP measurements of the central adhesions formed following inhibition of actomyosin contractility for 30 minutes were also taken, specifically to quantify the recovery parameters of EGFP-ILK (Figure S3). These measurements showed that ILK in these newly formed adhesions displayed k_on_ and k_off_ values that were essentially identical, though an order of magnitude lower, than those measured on FAs of untreated cells. Comparison of k_on_ and k_off_ values confirmed that in untreated FAs, ILK is in an “assembly state” (k_on_ > k_off_), but switches to a “disassembly state” (k_on_ < k_off_) following a few minutes of Y-27632 treatment; while in the novel central adhesions, ILK exists in a “stable state” (k_on_ = k_off_), even in the presence of the inhibitor.

## Discussion

In this study, we addressed a basic aspect of adhesion biology; namely, the molecular, structural and functional heterogeneity of integrin adhesions, and the mechanisms whereby their molecular variability is regulated by mechanical force. The diversity of integrin adhesions has been extensively documented in recent years, showing remarkable differences between the various classes of integrin-based ECM adhesions such as focal complexes, FAs, fibrillar adhesions and podosomes [4,5,15,36]. It was further demonstrated that different regions within individual adhesion sites can display distinct molecular compositions [37]. This molecular heterogeneity is not surprising, given the extraordinary complexity and diversity of the ECM itself, displaying complex and irregular molecular composition and mechanical properties [5,38–40]. Furthermore, the molecular machinery of the adhesion sites (known, collectively, as the “integrin adhesome”), comprising a large variety of receptors, adaptor molecules, cytoskeletal components and signaling proteins, can assemble into diverse complexes with distinct structural and functional properties [22]. In this study, we explored the possibility that the molecular properties of FAs are locally regulated, by actomyosin-driven contractile forces.

The general notion that FA assembly depends on local mechanical forces is well-established (e.g. [17,41–43]); yet the nature of the molecular cues whereby changes in local forces (“physical cues”) affect FA composition (“chemical response”), is still largely unclear. For example, it is not known whether contractile forces are needed to keep the FAs structure intact, and upon reduction in these forces, the entire structure falls apart, or whether the turnover of different FA components is individually regulated. Our main objective in this study was to dynamically track the fate of multiple adhesome components after shifting live cells from their normal, contractile state to a “relaxed” state, induced by Rho-kinase inhibition. Progressing stepwise from the structural-phenomenological level to the molecular-mechanistic level, we made the following observations:

1 Each of the eight proteins tested in this work dissociated from FAs at a different, characteristic rate, with zyxin and VASP being the fastest to exit, and kindlin-2 the slowest.
2 The subcellular location of the FAs had a marked effect on the rate of dissociation of all components, with “central adhesions” dissociating faster than those located at the cell periphery.
3 Structural analysis of FAs, based on cryo-ET, revealed major changes in the nanostructure of these adhesion sites, characterized by rapid disorganization of actin filaments shortly (<3 minutes) after addition of the inhibitory drug, suggesting that the loss of stress fiber-mediated tension precedes the dissociation of the adhesome molecules.
4 *De novo* formation of integrin adhesions with distinct subcellular distributions and molecular compositions takes place during and after the Y-27632-induced dissociation of the pre-existing FAs.
5 Rho-kinase inhibition had a differential effect on the molecular turnover kinetics (measured by FRAP) of the tested adhesome components. The apparent changes in k_on_ and k_off_ values and, hence, the predicted dissociation rates calculated for the different proteins following Y-27632 treatment, fit their measured dissociation rates.

A critical observation reported here is the distinct dynamic behavior of the different adhesome components tested, either in unperturbed cells, or in cells treated with Y-27632. These differences manifest themselves in: (1) different rates of dissociation from FAs following Rho-kinase inhibition; and (2) different turnover rates of the respective proteins in the presence and absence of the drug. These two features appear to be affected by mechanical stress: the major challenge of this work was to decipher the mechanistic interrelationships between the macroscopic observation (FA integrity) and the nanoscopic processes (e.g., turnover of specific proteins, or the organization of the actin filaments connected to the FA-associated particles).

It is noteworthy that the differential exit and entry of distinct FA proteins under different force regimes has been extensively documented in the past. Zamir et al. (2000) demonstrated that tensin and α_5_β_1_ integrin exit FAs to form fibrillar adhesions, leaving behind paxillin, vinculin, α_v_β_3_ integrin, and most of the tyrosine phosphorylated components of FAs. Rottner et al. (2001) reported on the recruitment of zyxin to new adhesion sites, yet its absence from lamellipodial and filopodial tips. They also noted that zyxin is an early target for signals leading to adhesion disassembly, in as much as it exits FAs before vinculin and paxillin, in line with the data reported here, and VASP dissociation occurs at an intermediate rate, between zyxin and vinculin/ paxillin. Small differences between their results and the findings reported here can be attributed to the choice of other cell types, and use of combined expression of constitutively active Rac1 and Y-27632, in contrast with Y-27632 alone, in our experiments. In a more recent study, by Pasapera et al (2010), differential recruitment and dissociation of FA proteins is described, including the involvement of myosin II-dependent contractility in these processes. The results reported here extend the scope of these studies by examining a considerably larger array of FA components, and go one important step further, towards understanding the mechanism underlying the differential kinetic behavior of the different proteins, by calculating the specific changes in k_on_ and k_off_ values that drive the turnover of each of the proteins, and their dissociation from FAs, upon Y-27632 treatment.

Mechanical force is a critical factor in the regulation of FA formation and stability. This notion is based on the well-documented dissociation of FAs upon treatment with Rho-kinase or actomyosin inhibitors [16,18,19,34,35,44]. However, the temporal order of the reduction in stress and loss of adhesome components has not been established. The cryo-ET images acquired following treatment with Y-27632 support the notion that Rho-kinase inhibition induces marked structural changes in the adhesion sites shortly after addition of the inhibitor, but prior to the apparent protein dissociation from FAs. This evidence corroborates our previous finding that reduction in traction force upon myosin II inhibition, deduced by observing wrinkles produced by HeLa cells plated onto a flexible silicone rubber substrate, is essentially instantaneous, occurring in less than 20 seconds [25]. Y-27632-mediated effects manifest themselves in early loss of alignment of actin filaments (Figure 4 and [29]), and appear to be related to a reduction in the level of mechanical stress applied by the acto-myosin system.

Once the forces applied to the adhesion sites are reduced, a general dissociation of FAs takes place, characterized by differential exit rates of various adhesome molecules. Attempts to understand the mechanism underlying this phenomenon indicated that the relaxation of Rho kinase-dependent actomyosin contractility radically and differentially affects the k_on_ and k_off_ values (calculated based on FRAP analyses) of the different proteins, in the presence and absence of Y-27632. In general, an increase in k_off_ over k_on_ values was seen, inevitably inducing net dissociation of each of the tested proteins from the adhesion sites. We further demonstrated that the effects of Y-27632 on the k_off_ and k_on_ values were highly protein-specific, leading to different predicted dissociation rates of the various proteins tested. Importantly, these predicted dissociation values were in excellent agreement with those actually measured by quantitative live-cell microscopy, supporting the notion that the mechanosensitivity of FAs may be attributed to differential, force-dependent regulation of the assembly-disassembly kinetics of individual FA components. Notably, although there is a similar trend among the calculated and measured disassembly curves for the different proteins, the calculated disassembly rates were faster than those actually measured. The reason could be that the disassembly process depends both on diffusion and exchange between membrane-bound and cytoplasmic proteins. Therefore, describing the process as dependent only on the exchange rate constants is an approximation. Nevertheless, the remarkable similarity between the measured and calculated curves suggests that exchange is the more dominant process in adhesion disassembly. Incorporating all dynamic processes into a model that describes FA disassembly is a focus for future efforts.

Another observation reported here is the apparent difference between integrin adhesions formed following Y-27632 treatment, and FAs of untreated cells. In general, the assembly of adhesion sites was shown to be highly dependent on actin organization, even in structures devoid of actomyosin contractility, e.g., small adhesions near the leading edge were reported to transmit strong traction forces in an actin polymerization-dependent but myosin II-independent manner [44–47]. Apparently, peripheral FAs, formed in the presence of Y-27632, display markedly reduced levels of zyxin and kindlin-2 (see Figure 5). It is noteworthy that zyxin accumulation in adhesion sites in a Rho-kinase- and myosin II-mediated, force-dependent manner was previously reported [25,35,45–47], yet mechanosensitive recruitment of kindlin-2 has not been previously described.

The integrin adhesions formed in the central regions of Y-27632-treated HeLa cells display limited association with F-actin (in contrast to α_V_ integrin-based adhesions reported by [48]), as well as a unique molecular composition, with prominent levels of α_V_ integrin, ILK, kindlin-2 and paxillin, the adhesome components known to strongly interact with one another [49–53]; and little or no vinculin, talin, FAK, zyxin and VASP. It is interesting to note that FRAP experiments involving ILK in these central adhesions indicated that the k_on_ and k_off_ values were essentially the same, which would explain their stability in the presence of the drug.

In conclusion, this study highlights the molecular and structural diversity of integrin adhesions, and demonstrates the central role of mechanical forces in regulating this diversity. Quantifying eight different adhesome molecules, we show that integrin adhesions display diverse "molecular signatures", and that mechanical forces can play a key role in regulating FA composition. We further propose a novel mechanism underlying this mechanosensitivity, in which forces applied to the adhesion site selectively modulate the on- and off-rates of different FA constituents, thereby regulating the molecular composition of FAs, and thus, their stability and fate.

## Supporting Information

Figure S1 **Correlative fluorescence and cryo-ET microscopy.** Correlated microscopy, combining fluorescence microscopy and cryo-electron tomography, was used to study the effect of Y-27632 on actin organization in REF-52 cells expressing YFP-tagged paxillin. Cells growing on EM grids were first examined by fluorescence light microscopy, then transferred to the cryo-EM, and specific FAs were identified (white border). Cryo-electron tomograms of the adhesion sites were collected. The inset shows the adhesion site (black arrow) at low magnification cryo-EM.
(TIF)

Figure S2 **Effect of Y-27632 treatment on FA reorganization in REF52 cells.** Untreated cells (control) and cells treated with 10 µM Y-27632 for 3,10 and 30 min were stained for actin, paxillin and vinculin, demonstrating that the spatio-temporal dynamics of FAs in response to Y-27632 are similar to those of HeLa JW cells (Figure 1).
(TIF)

Figure S3 **k_on_ vs. k_off_ values of GFP-ILK at different adhesion sites.** FRAP measurements were performed either on untreated HeLa cells, within 10 minutes of their treatment with Y-27632 (initial FAs), or after 30 minutes of such treatment (newly formed central adhesions). The resulting k_on_ and k_off_ values ± SEM are presented. Note that in the new adhesions, k_on_ > k_off_, indicating a new steady-state, though with slower kinetics than that of ILK in untreated cells.
(TIF)
