# Correction: Differential Effect of Actomyosin Relaxation on the Dynamic Properties of Focal Adhesion Proteins

**DOI:** 10.1371/journal.pone.0090269

**Published:** 2014-02-18

**Authors:** The PLOS ONE Staff

A funding organization and grant were incorrectly omitted from the Funding statement. The Funding statement should read: "This study was supported by the National Institutes of Health (NIH) Common Fund Nanomedicine Program (PN2 EY016586), an NoE Systems Microscopy Grant no. 258068, funded through the European Union’s Seventh Framework Programme (to BG), an ERC Starting Grant 243047 INCEL (to OM), and a Deutsche Forschungsgemeinschaft-Deutsch-Israelische Projektkooperation grant (KL 1948/1-1, GA 309/10-1) (to YIH). The funders had no role in study design, data collection and analysis, decision to publish or preparation of the manuscript."
