# Supplementary figures and images for: Differential Effect of Actomyosin Relaxation on the Dynamic Properties of Focal Adhesion Proteins
Source: PLoS One. 2013 Sep 9;8(9):e73549. doi: 10.1371/journal.pone.0073549 (PMC3767655; doi:10.1371/journal.pone.0073549)

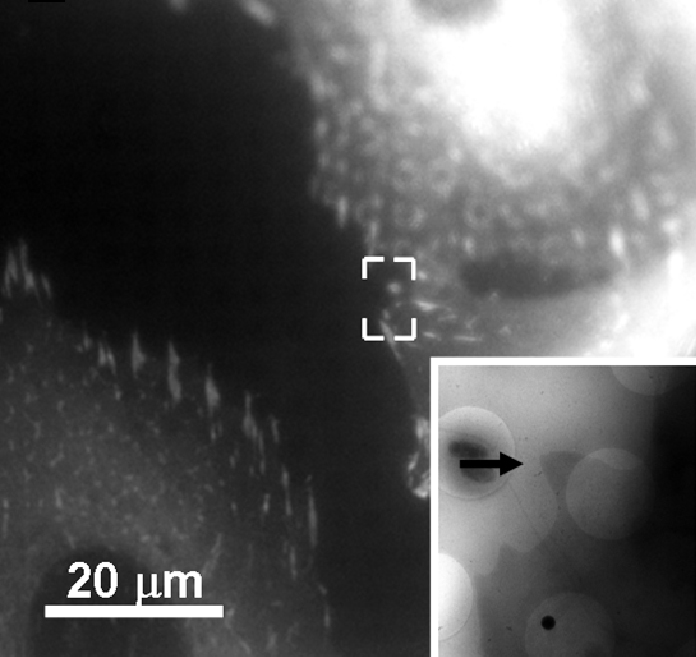

Supplement: Figure S1 — Correlative fluorescence and cryo-ET microscopy. Correlated microscopy, combining fluorescence microscopy and cryo-electron tomography, was used to study the effect of Y-27632 on actin organization in REF-52 cells expressing YFP-tagged paxillin. Cells growing on EM grids were first examined by fluorescence light microscopy, then transferred to the cryo-EM, and specific FAs were identified (white border). Cryo-electron tomograms of the adhesion sites were collected. The inset shows the adhesion site (black arrow) at low magnification cryo-EM. (TIF) [file pone.0073549.s001.tif]

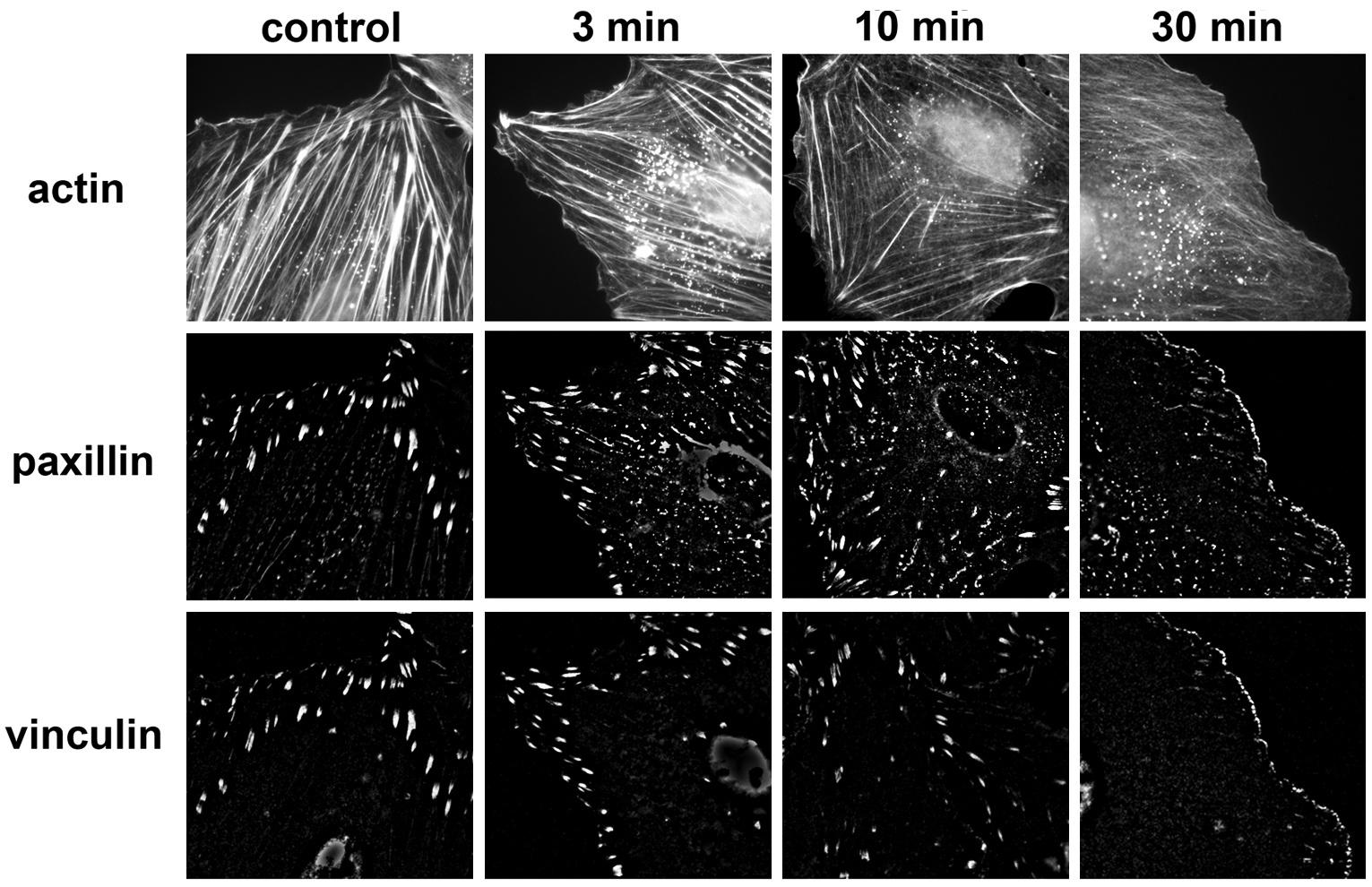

Supplement: Figure S2 — Effect of Y-27632 treatment on FA reorganization in REF52 cells. Untreated cells (control) and cells treated with 10 µM Y-27632 for 3,10 and 30 min were stained for actin, paxillin and vinculin, demonstrating that the spatio-temporal dynamics of FAs in response to Y-27632 are similar to those of HeLa JW cells (Figure 1). (TIF) [file pone.0073549.s002.tif]

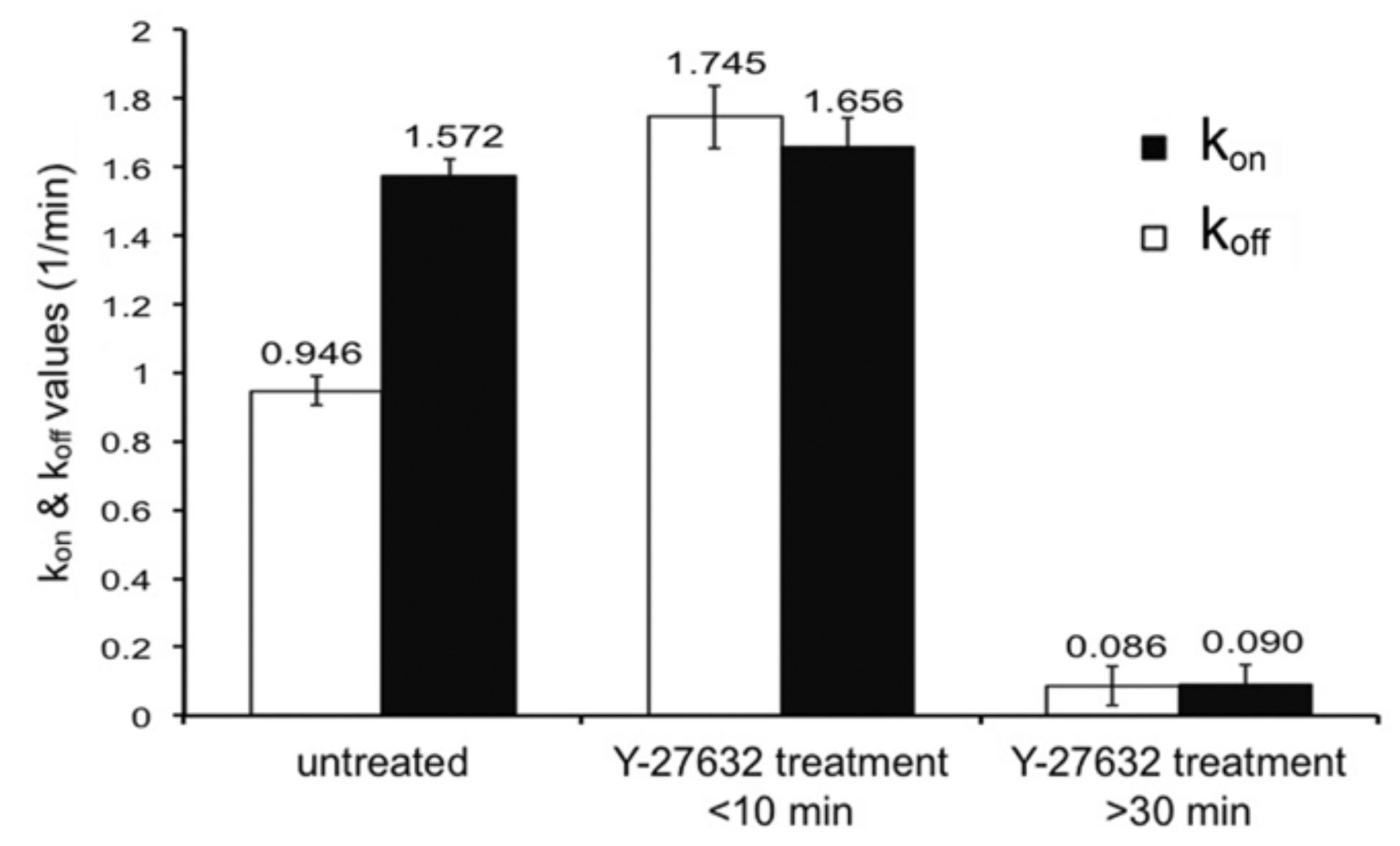

Supplement: Figure S3 — kon vs. koff values of GFP-ILK at different adhesion sites. FRAP measurements were performed either on untreated HeLa cells, within 10 minutes of their treatment with Y-27632 (initial FAs), or after 30 minutes of such treatment (newly formed central adhesions). The resulting kon and koff values ± SEM are presented. Note that in the new adhesions, kon > koff, indicating a new steady-state, though with slower kinetics than that of ILK in untreated cells. (TIF) [file pone.0073549.s003.tif]
